# Resilience in family caregivers of patients diagnosed with advanced cancer – unravelling the process of bouncing back from difficult experiences, a hermeneutic review

**DOI:** 10.1080/13814788.2020.1784876

**Published:** 2020-07-07

**Authors:** Sophie Opsomer, Jan De Lepeleire, Emelien Lauwerier, Peter Pype

**Affiliations:** a Academisch Centrum voor Huisartsgeneeskunde (ACHG), Catholic University Leuven, Leuven, Belgium; b Department of Public Health and Primary Care, Ghent University, Ghent, Belgium; c Department of Experimental Clinical and Health Psychology, Ghent University, Ghent, Belgium; d End-of-Life Care Research Group, VUB and Ghent University, Ghent, Belgium

**Keywords:** Palliative and terminal care, general practice/family medicine, general, psychological problems

## Abstract

**Background:**

Despite the risk for developing mental disorders, most of advanced cancer patients’ family caregivers undergo a resilient process throughout the caregiving period. Research on resilience in caregivers of advanced cancer patients is scarce and further hindered by the lack of a univocal definition and a theoretical framework.

**Objectives:**

To provide clarity on the concept of resilience by proposing an integrative view that can support health care professionals and researchers in conducting and interpreting research on resilience.

**Methods:**

The review process was inspired by the hermeneutic methodology: a cyclic review process, consisting of repeated searching and analysing until data saturation is reached and focussed on achieving a deeper understanding of ill-defined concepts. The definitions from eighteen reviews on resilience and the theoretical frameworks from eight concept analyses were analysed. The composing elements of resilience were listed and compared.

**Results:**

The American Psychological Association’s definition of resilience and Bonanno’s theoretical framework are suggested to guide further research on resilience. Moreover, four knowledge gaps were uncovered: (1) How do resilience resources interact? (2) What are the key predictors for a resilient trajectory? (3) How do the resilient trajectories evolve across the caregiving period? And (4) how does the patient’s nearing death influence the caregiver’s resilience?

**Conclusion:**

To address flaws in conceptualisation and the resulting gaps in knowledge, we suggest a definition and a theoretical framework that are suited to allow heterogeneity in the field, but enables the development of sound interventions, as well as facilitate the interpretation of intervention effectiveness.

KEY MESSAGES

- Research on resilience in general and in cancer caregiving in particular is hampered by the lack of a universally accepted definition and a theoretical framework.
- A hybrid approach drawing on the American Psychological Association’s definition and Bonanno’s framework offers a guide for the study of resilience in caregivers of advanced cancer patients.

## Introduction

Homecare for a family member diagnosed with advanced cancer often comes with significant burden [1]. Being diagnosed with incurable cancer can be considered a potentially traumatic event (PTE) for both the patient and their family caregivers (relatives, neighbours, or friends, who take up the caregiving role without being paid) [2]. Hence, some caregivers may be at risk of psychological, physical and social dysfunction (e.g. major depression, anxiety, fatigue, sleeplessness or social isolation) [3], while others will not experience the diagnosis as a traumatic event [4]. However, after a short period of disruption, a significant group will probably follow a resilient process, characterised by recovery to a status of healthy functioning, or will even find benefits in caregiving [5–7].

Although emotional distress is generally higher in family members than in patients, family caregivers often fail to seek medical help for themselves [8]. Difficulties arise for healthcare professionals in timely identification of those family caregivers at risk of severe mental disabilities from those who follow a resilience trajectory. Hence, medical and psychological help often comes too late to prevent any mental disruption, which not only affects the caregiver but also influences the patient’s well-being [9].

Most interventions in caregivers are oriented towards prevention of anxiety or depression. However, only minor and temporary effects are registered [10, 11]. Interventions in other populations, focussing on strengths and protective factors endorsing a person’s resilience seem to be more promising [12,13]. Nevertheless, due to the situation-specificity of resilience, those interventions cannot be applied as such to family caregivers of cancer patients.

Studies on resilience in caregivers of advanced cancer patients are scarce [4,6,7,14], and the information needed to develop a resilience-supporting intervention in primary cancer care is still lacking. Hence, more research in this field is vital. However, the progression of studies on resilience is critically hampered by some ambiguities. There is, for instance, no universally accepted definition of resilience, so it has been conceptualised in different ways and circumstances. Although heterogeneity may not necessarily be an issue, it has been observed that those conceptual discrepancies have led to a variety of study designs and resilience scales which seriously hinder the interpretation and comparison of study results [15,16].

This study aims to bring clarity to researchers for further exploration and a better understanding of resilience in caregivers of advanced cancer patients by answering the following research questions:

- Which elements consistently arise from the definitions of resilience following a PTE? What definition is the most comprehensive and could be suggested for further research?
- Which existing theoretical framework of resilience following a PTE is the most comprehensive, could enhance methodological soundness, and could be suggested for further research in cancer caregiving?

## Methodology

The objective of this review is to advance theoretical understanding of the concept of resilience following a PTE by a critical reflection of existing definitions and frameworks. The methodology suggested for this purpose is a hermeneutic review [17,18]. Hermeneutics is a methodology suited to conducting a literature review that aims to explore and to clarify a vague or ill-defined concept. The hermeneutic process is cyclical and requires researchers to move repeatedly through a circle of searching for existing literature through different databases and analysing the included articles until data saturation is reached [18].

Between February and May 2019, a hermeneutic, circular search strategy was applied [18]. Four databases (PubMed, Embase, Cinahl, and PsycInfo) were searched for reviews - systematic reviews, background papers, narrative reviews - and concept analyses. No time frame for literature search was applied. Search terms related to resilience, potentially traumatic events, adults, advanced cancer and caregivers were applied in different combinations. After an orientating reading and a first analysis of the selected articles, a new search-cycle was initiated with additional search terms. This process was repeated several times, until no new elements were found in either definitions or frameworks. Intermediate results were discussed within the team.

Next, the definitions were compared, and similarities listed. Subsequently, we searched for a definition that comprised the repeated elements.

In the final step, the resulting frameworks from the included concept analyses were listed. Theoretical frameworks are essential in making research findings more meaningful and to ground them into existing theories about a complex concept [19]. An appropriate framework should be broad and comprehensive, comprising the most elements from the theories about resilience. For this study, the framework should also be applicable or adaptable to the situation of a caregiver of a patient with advanced cancer.

An overview of the background and expertise of the authors and their contribution to the review is annexed as Supplementary material.

## Results and discussion

### Definitions of resilience in adults exposed to a PTE

There is little or no consensus around the terminology used to define resilience. Rutter describes resilience as a positive pole of the response to adversity [20], Glantz and Johnson [21], Masten [22], Fergus and Zimmerman [23], and Seery and Quinton consider resilience as an outcome [24], and Fraser attributes resilience as an ability [25]. Most authors, however, define resilience as a process [26–28]. This diversity of definitions reflects the variation of ways of looking at resilience across context and situation and underscores the multidimensionality and the complexity of the concept. However, uniformity in definition and conceptualisation of resilience would add to the validity, reliability, comparability and transferability of study results.

Comparing the definitions proposed in the reviewed publications (presented as Supplementary material) [2,12,24,28–42] the following elements repeatedly arise:

- Resilience is a dynamic process that can be developed or learned [36,38,40–42].
- Resilience starts from exposure to a PTE (e.g., adversity, threat, stressful event or adverse life event) and is related to the experience [2,12,24,30–39,41].
- During a resilient trajectory, positive adaptation to a PTE is achieved despite experienced difficulties or disruptive events [12,24,33,38].
- There is either a neutral or a positive outcome in response to the PTE, such as healthy functioning [2,28,30,32,38,39], bouncing back [12,33,36,41] or finding benefits [31,35].

Result: All of these elements are reflected in the definition of the American Psychological Association (APA) (slightly rephrased in Yehuda’s review [12,43]): *‘Resilience is the process of adapting well in the face of adversity, trauma, tragedy, threats or significant sources of stress – such as family and relationship problems, serious health problems or workplace and financial stressors. It means "bouncing back" from difficult experiences.’*

### Concept analyses and their resulting theoretical frameworks

To study resilience, the definition put forward by the APA is very useful. However, a more comprehensive framework is needed to unravel the complex nature of the interacting elements in the process of bouncing back.

#### Conceptualisation of resilience

Resilience has been conceptualised in different ways.

- Resiliency or ego-resiliency considers resilience as a personality trait or a person’s resilience resources and does not guarantee a resilient process [27,29,44].
- Resilience as a biopsychospiritual homeostasis describes how the interaction between the protective, resilient attributes and the threats which accompany adversity can lead to biopsychospiritual homeostasis on the one hand or to dysfunction in people who lack resilient qualities on the other [44].
- Recently, most experts agree that resilience is a dynamic process that cannot be considered separately from the potentially traumatic event (PTE) [2,28,32,42,45–49]. (More detailed representations of the conceptualisation and the history are annexed as Supplementary material)

#### Resilience as a process following a PTE

From the frameworks resulting from the reviewed concept-analyses (presented as Supplementary material) [16,33,36,44,49–52], some key features about resilience following a PTE emerge:

- Richardson’s and Bonanno’s frameworks highlight the resilient outcome [44,49]. Resilience can be acquired through exposure to stressors or adversity and can change over time [44,47].
- Most frameworks underline the need for specific individual characteristics and coping styles [16,33,36,49–52].
- All frameworks emphasise the importance of the association between a PTE and the resilience process [16,33,36,44,49–52]. Resilience is highly situation-related, meaning that a person who seems to cope adaptively in one situation can fail to adapt well in another case [47]. Hence, resilience should always be approached within a specific context.
- Both the theoretical frameworks of Liu and Bonanno underscore the importance of situating people within their broader socio-economic context [49,52].
- Most frameworks point to the dynamics of the resilience process (e.g., development through learning from earlier PTEs or reinforcement by mutual interactions) [16,36,44,49,50,52]. Each context consists of different levels that all act and interact with each other, and in the end, can be resilience-supporting or resilience-threatening [26,47].

Result: From our review, we consider Bonanno’s framework ‘temporal elements of resilience’ the most suitable to study resilience following a PTE in general and in caregivers of advanced cancer patients in particular [49]. The framework can be considered the most comprehensive as it combines all four elements of resilience reflected in the APA definition as well as all aspects of resilience from the concept analyses (Box 1).

BOX 1. Suggested definition and framework

The APA definition of resilience [43]: *‘Resilience is the process of adapting well in the face of adversity, trauma, tragedy, threats or significant sources of stress – such as family and relationship problems, serious health problems or workplace and financial stressors. It means ‘bouncing back’ from difficult experiences.’* Bonanno’s theoretical framework on resilience comprises four elements [49]:
1. The essential association with the PTE encompassing short- and long-term exposure.
2. The pre-adversity capacity to adjust.
3. The post-adversity resilient outcomes that are more than merely the absence of disease or average-level adjustment.
4. The predictors of a resilient outcome resulting from the amplified interactions and reciprocal processes within an array of individual and social variables.

#### Resilience in cancer caregiving: state of the art and gaps in knowledge

Very few studies addressing resilience in adult caregivers of adult cancer patients have been published [4,6,7]. However limited, these studies enhance the knowledge and insight into the intrinsic and extrinsic resources that either facilitate or hamper resilience after having lost a family member diagnosed with advanced cancer. In their qualitative study, Opsomer et al. report on how the caregivers’ pre-adversity capacity can lead to a resilient process throughout caregiving, characterised by a positive outcome [4]. The study findings fit into Bonanno’s framework as follows: The resilient processes were facilitated by interacting, intrinsic resources (adaptive flexibility, positivism, a sense of self-initiative and adaptive dependency) and context-related resources (availability of the context, meaningful relationships and the quality of the marital relationship).

Nevertheless, applying Bonanno’s framework to existing research also reveals four important knowledge gaps, two in research on resilience in general and two in research applied to resilience in cancer caregiving specifically.

1. Little is known about how resilience resources interact.
2. It is not clear what the key predictor variables for a resilient trajectory are.
3. The diagnosis of advanced cancer, which can be considered a PTE, is seldom followed by a period of stability. Mostly, this period is dominated by repetitive, stressful events (e.g., hospital admissions, financial problems or recurrent bad news). To the best of our knowledge, no studies have been published that shed light on the resilience trajectories and how they evolve across the caregiving period from diagnosis to death of the patient.
4. Indicators of nearing death, such as anorexia, severe weight loss, or reduced consciousness, are all events that require coping, and hence, could interfere with a resilient trajectory [24,53,54]. As far as we know, the influence of those stressors on a caregiver’s resilience, has not yet been studied.

#### Strengths and limitations

When it comes to a deeper understanding of a complex and ill-defined concept, a hermeneutic review is preferred over a classic systematic review. The cyclical hermeneutic approach of this review guarantees a thorough search and a critical reflection on the dataset. Moreover, the perpetual cycle of searching, analysing and searching again with new search terms until data-saturation is reached, would turn up articles that traditionally would have remained hidden through a predefined search string [17,18].

This study was carried out using a two-step approach in which definitions and theoretical frameworks of resilience following a PTE were compared and analysed. Subsequently, these findings were verified for the particular situation of advanced cancer caregiving.

The primary study limitations lie within the restriction of the search by reviews and concept analyses, meaning that supplementary elements from experts’ individual definitions formulated in original papers were dismissed.

Moreover, theoretical frameworks on unmet needs and the vulnerability of caregivers of advanced cancer patients were not reviewed. Nevertheless, they could provide important valuable information and could be of interest to improve cancer-caregiver supporting programmes.

#### Implications for research

In this review, we advocate the use of the APA definition and Bonanno’s theoretical framework for further research on resilience following a PTE in general and on resilience in advanced cancer caregiving in particular. Both the definition and framework can be broadly implemented.

It is known that eliminating conceptual heterogeneity in the definitions could facilitate the interpretation of study results and the development of interventions aimed at promoting resilience. Moreover, the use of one definition and transparency in the framework that is applied, would add to the transferability, validity, reliability and comparability of the study results.

So far, to overcome conceptual heterogeneity, resilience has often been looked at as an absence of depression, post-traumatic stress disorder or traumatic grief [39,45,48,55]. However, to fill the gaps in knowledge on resilience in general, as they are listed above, resilience should be explored to its full extent since resilience encompasses more than merely absence of disease [49]. Such a multifaceted approach needs a clear definition and is facilitated by the use of a comprehensive framework. Even so, filling the gaps in the knowledge of resilience in cancer caregiving could be facilitated by the consistent use of the APA definition and Bonanno’s framework as in this way, the use of a variety of labels for the same concept [56] could be avoided.

To detect caregivers at risk for a major psychosocial dysfunction and to redirect them in time to a resilient trajectory, insight into the resilience trajectories and critical turn-over points is paramount. However, findings can only be interpreted correctly and translated to practice if the definition and framework used in research are delineated.

## Conclusion

There is a vital need for further research on resilience in caregivers of advanced cancer patients. Focussing on resilience could aid in identifying those caregivers at risk for mental disorders earlier and could advance the development of innovative prevention programmes and treatment options.

To overcome the difficulties encountered in resilience research, we suggest that researchers should clearly define resilience and explain the theoretical framework on which the research is built. Additionally, a universally accepted definition and theoretical framework is desirable. For this purpose, we suggest the APA definition of resilience and Bonanno’s framework ‘the temporal elements of resilience’ [43,49].

## Supplementary Material

Supplemental Material - Research on resilience: history

Supplemental Material - Conceptualization of resilience

Supplemental Material - Author contributions

Supplemental Material - Theoretical frameworks of resilience

Supplemental Material - Definitions of resilience

## References

[1] Goren A, Gilloteau I, Lees M, et al. Quantifying the burden of informal caregiving for patients with cancer in Europe. Support Care Cancer. 2014;22(6):1637–1646. 24496758 10.1007/s00520-014-2122-6

[2] Bonanno GA. Loss, trauma, and human resilience: have we underestimated the human capacity to thrive after extremely aversive events? Am Psychol. 2004;59(1):20–28. 14736317 10.1037/0003-066X.59.1.20

[3] Trevino KM, Prigerson HG, Maciejewski PK. Advanced cancer caregiving as a risk for major depressive episodes and generalized anxiety disorder. Psycho Oncol. 2018;27(1):243–249. 10.1002/pon.4441 PMC5746474 28426918

[4] Opsomer S, Pype P, Lauwerier E, et al. Resilience in middle-aged partners of patients diagnosed with incurable cancer: a thematic analysis. PloS One. 2019;14(8):e0221096. 31412074 10.1371/journal.pone.0221096 PMC6693771

[5] Cassidy T. Benefit finding through caring: the cancer caregiver experience. Psychol Health. 2013;28(3):250–266. 22928621 10.1080/08870446.2012.717623

[6] Roen I, Stifoss-Hanssen H, Grande G, et al. Resilience for family carers of advanced cancer patients-how can health care providers contribute? A qualitative interview study with carers. Palliat Med. 2018;32(8):1410–1418. 29852808 10.1177/0269216318777656

[7] Hwang IC, Kim YS, Lee YJ, et al. Factors associated with caregivers' resilience in a terminal cancer care setting. Am J Hosp Palliat Care. 2018;35(4):677–683. 29141459 10.1177/1049909117741110

[8] Li Q, Loke AY. A spectrum of hidden morbidities among spousal caregivers for patients with cancer, and differences between the genders: a review of the literature. Eur J Oncol Nurs. 2013;17(5):578–587. 23465710 10.1016/j.ejon.2013.01.007

[9] Kershaw T, Ellis KR, Yoon H, et al. The interdependence of advanced cancer patients' and their family caregivers' mental health, physical health, and self-efficacy over time. Ann Behav Med. 2015;49(6):901–911. 26489843 10.1007/s12160-015-9743-y PMC4825326

[10] O'Toole MS, Zachariae R, Renna ME, et al. Cognitive behavioral therapies for informal caregivers of patients with cancer and cancer survivors: a systematic review and meta-analysis. Psychooncology. 2017;26(4):428–437. 27147198 10.1002/pon.4144 PMC5412844

[11] Griffin JM, Meis LA, MacDonald R, et al. Effectiveness of family and caregiver interventions on patient outcomes in adults with cancer: a systematic review. J Gen Intern Med. 2014;29(9):1274–1282. 24841557 10.1007/s11606-014-2873-2 PMC4139519

[12] Yehuda R, Flory JD, Southwick S, et al. Developing an agenda for translational studies of resilience and vulnerability following trauma exposure. Ann N Y Acad Sci. 2006;1071:379–396. 16891584 10.1196/annals.1364.028

[13] Joyce S, Shand F, Tighe J, et al. Road to resilience: a systematic review and meta-analysis of resilience training programmes and interventions. BMJ Open. 2018;8(6):e017858. 10.1136/bmjopen-2017-017858 PMC6009510 29903782

[14] Saria MG, Courchesne N, Evangelista L, et al. Cognitive dysfunction in patients with brain metastases: influences on caregiver resilience and coping. Support Care Cancer. 2017;25(4):1247–1256. 27921222 10.1007/s00520-016-3517-3 PMC10187463

[15] Fletcher D, Sarkar M. Psychological resilience. A review and critique of definitions, concepts and theory. Eur Psychol. 2013;18(1):12–23.

[16] Davydov DM, Stewart R, Ritchie K, et al. Resilience and mental health. Clin Psychol Rev. 2010;30(5):479–495. 20395025 10.1016/j.cpr.2010.03.003

[17] Greenhalgh T, Thorne S, Malterud K. Time to challenge the spurious hierarchy of systematic over narrative reviews? Eur J Clin Invest. 2018;48(6):e12931. 29578574 10.1111/eci.12931 PMC6001568

[18] Boell SK, Cecez-Kecmanovic D. A hermeneutic approach for conducting literature reviews and literature searches. Commun Assoc Inf Syst. 2014;34:257–286.

[19] Dickson A, Emad K, Hussein , Joe AA. Theoretical and conceptual framework: mandatory ingredients of a quality research. Int J Sci Res. 2018;7:438–441.

[20] Rutter M. Psychosocial resilience and protective mechanisms. Am J Orthopsychiatry. 1987;57(3):316–331. 3303954 10.1111/j.1939-0025.1987.tb03541.x

[21] Glantz MD, Johnson JL. Resilience and development: positive life adaptations. In: Glantz MD, Johnson JL, editors. Longitudinal research in the social and behavioral sciences: an interdisciplinary series. New York (NY): Kluwer Academic/Plenum Publishers; 1999.

[22] Masten AS. Ordinary magic. Resilience processes in development. Am Psychol. 2001;56(3):227–238. 11315249 10.1037//0003-066x.56.3.227

[23] Fergus S, Zimmerman MA. Adolescent resilience: a framework for understanding healthy development in the face of risk. Annu Rev Public Health. 2005;26:399–419. 15760295 10.1146/annurev.publhealth.26.021304.144357

[24] Seery MD, Quinton WJ. Understanding resilience: from negative life events to everyday stressors. Adv Exp Soc Psychol. 2016;54:181–245.

[25] Fraser MW, Richman JM, Galinsky MJ. Risk, protection, and resilience: towards a conceptual framework for social work practice. Soc Work Res. 1999;23:129–208.

[26] Kumpfer KL. Factors and processes contributing to resilience. The resilience framework. In: Johnson GA, editor. Resilience and Development. New York: Kluwer Academic/Plenum Publishers; 1999. p. 179–224.

[27] Luthar SS, Cicchetti D, Becker B. The construct of resilience: a critical evaluation and guidelines for future work. Child Dev. 2000;71(3):543–562. 10953923 10.1111/1467-8624.00164 PMC1885202

[28] Bonanno GA. Resilience in the face of potential trauma. Curr Dir Psychol Sci. 2005;14(3):135–138.

[29] Letzring TD, Block J, Funder DC. Ego-control and ego-resiliency: generalization of self-report scales based on personality descriptions from acquaintances, clinicians, and the self. J Res Personal. 2005;39(4):395–422.

[30] Bonanno GA, Mancini AD. The human capacity to thrive in the face of potential trauma. Pediatrics. 2008;121(2):369–375. 18245429 10.1542/peds.2007-1648

[31] Grafton E, Gillespie B, Henderson S. Resilience: the power within. Oncol Nurs Forum. 2010;37(6):698–705. 21059582 10.1188/10.ONF.698-705

[32] Bonanno GA, Westphal M, Mancini AD. Resilience to loss and potential trauma. Annu Rev Clin Psychol. 2011;7:511–535. 21091190 10.1146/annurev-clinpsy-032210-104526

[33] Windle G. What is resilience? A review and concept analysis. Rev Clin Gerontol. 2011;21(2):152–169.

[34] Bonanno GA. Uses and abuses of the resilience construct: loss, trauma, and health-related adversities. Soc Sci Med. 2012;74(5):753–756. 22300714 10.1016/j.socscimed.2011.11.022

[35] Rutter M. Resilience as a dynamic concept. Dev Psychopathol. 2012;24(2):335–344. 22559117 10.1017/S0954579412000028

[36] Garcia-Dia MJ, DiNapoli JM, Garcia-Ona L, et al. Concept analysis: resilience. Arch Psychiatr Nurs. 2013;27(6):264–270. 24238005 10.1016/j.apnu.2013.07.003

[37] Hu T, Zhang D, Wang J. A meta-analysis of the trait resilience and mental health. Personal Individ Differ. 2015;76:18–27.

[38] Kalisch R, Baker DG, Basten U, et al. The resilience framework as a strategy to combat stress-related disorders. Nat Hum Behav. 2017;1(11):784–790. 31024125 10.1038/s41562-017-0200-8

[39] Galatzer-Levy IR, Huang SH, Bonanno GA. Trajectories of resilience and dysfunction following potential trauma: A review and statistical evaluation. Clin Psychol Rev. 2018;63:41–55. 29902711 10.1016/j.cpr.2018.05.008

[40] Rosa F, Bagnasco A, Aleo G, et al. Resilience as a concept for understanding family caregiving of adults with Chronic Obstructive Pulmonary Disease (COPD): an integrative review. Nurs Open. 2017;4(2):61–75. 28286662 10.1002/nop2.63 PMC5340167

[41] Autio T, Rissanen S. Positive emotions in caring for a spouse: a literature review. Scand J Caring Sci. 2018;32(1):45–55. 28543793 10.1111/scs.12452

[42] Eicher M, Matzka M, Dubey C, et al. Resilience in adult cancer care: an integrative literature review. Oncol Nurs Forum. 2015;42:3–16. 10.1188/15.ONF.E3-E16 25542332

[43] APA. The Road to Resilience. Washington (DC): The American Psychological Association (APA); [cited 2020 June 16]. Available from: http://www.apa.org/helpcenter/road-resilience.aspx

[44] Richardson GE. The metatheory of resilience and resiliency. J Clin Psychol. 2002;58(3):307–321. 11836712 10.1002/jclp.10020

[45] Bonanno GA, Kennedy P, Galatzer-Levy IR, et al. Trajectories of resilience, depression, and anxiety following spinal cord injury. Rehabil Psychol. 2012;57(3):236–247. 22946611 10.1037/a0029256

[46] Bonanno GA, Malgaroli M. Trajectories of grief: comparing symptoms from the DSM-5 and ICD-11 diagnoses. Depress Anxiety. 2020;37(1):17–25. Available from: https://onlinelibrary.wiley.com/doi/epdf/10.1002/da.22902 31012187 10.1002/da.22902

[47] Southwick SM, Bonanno GA, Masten AS, et al. Resilience definitions, theory, and challenges: interdisciplinary perspectives. Eur J Psychotraumatol. 2014;5. DOI:10.3402/ejpt.v5.25338 PMC4185134 25317257

[48] Mancini AD, Sinan B, Bonanno GA. Predictors of prolonged grief, resilience, and recovery among bereaved spouses. J Clin Psychol. 2015;71(12):1245–1258. 26394308 10.1002/jclp.22224

[49] Bonanno GA, Romero SA, Klein SI. The temporal elements of psychological resilience: an integrative framework for the study of individuals, families, and communities. Psychol Inq. 2015;26(2):139–169.

[50] Agaibi CE, Wilson JP. Trauma, PTSD, and resilience: a review of the literature. Trauma Violence Abuse. 2005;6(3):195–216. 16237155 10.1177/1524838005277438

[51] Gillespie BM, Chaboyer W, Wallis M. Development of a theoretically derived model of resilience through concept analysis. Contemp Nurse. 2007;25(1–2):124–135. 17622996 10.5172/conu.2007.25.1-2.124

[52] Liu JJW, Reed M, Girard TA. Advancing resilience: an integrative, multi-system model of resilience. Pers Individ Diff. 2017;111:111–118.

[53] Opsomer S, Joossens S, De Wit C, et al. Couples coping with nutrition-related problems in advanced cancer: a qualitative study in primary care. Eur J Oncol Nurs. 2019;38:76–84. 30717940 10.1016/j.ejon.2018.12.006

[54] Opsomer S, Joossens S, De Wit C, et al. Losing health symbols because of nutrition-related problems in advanced cancer: an interpretative phenomenological analysis. J Hosp Palliat Nurs. 2018;20(5):492–499. 30188444 10.1097/NJH.0000000000000471

[55] Bonanno GA, Wortman CB, Lehman DR, et al. Resilience to loss and chronic grief: a prospective study from preloss to 18-months postloss. J Pers Soc Psychol. 2002;83(5):1150–1164. 12416919 10.1037//0022-3514.83.5.1150

[56] Walshe C, Roberts D, Appleton L, et al. Coping well with advanced cancer: a serial qualitative interview study with patients and family Carers. PloS One. 2017;12(1):e0169071. 28107352 10.1371/journal.pone.0169071 PMC5249149

