# Supplementary material for: Resilience in family caregivers of patients diagnosed with advanced cancer – unravelling the process of bouncing back from difficult experiences, a hermeneutic review
Source: Eur J Gen Pract. 2020 Jul 7;26(1):79–85. doi: 10.1080/13814788.2020.1784876 (PMC7470057; doi:10.1080/13814788.2020.1784876)
Supplement: Supplemental Material - Research on resilience: history [file IGEN_A_1784876_SM1282.docx]

# Research on resilience: history

Interest in resilience research has grown notably in the last decade, and the number of publications per year continues to rise. Increasing attention has been paid to resilience along with the development of the ‘positive psychology’ movement. As a result, a problem-oriented approach emphasizing the maladaptive shifted to a strength-oriented approach, thus highlighting the positive [1, 2].

In 1824, Webster [3] described resilience as ‘*the ability of a stressed body to recover from or adjust easily to misfortune and change*’. Initial studies regarding resilience in disadvantaged children and adolescents raised under difficult circumstances identified certain characteristics, all of whom overcame the adversity of their situations [4-7].

During the twentieth century, not showing prolonged distress following a PTE or loss, was considered either pathological or exceptionally healthy. However, from recent research oriented towards positive aspects, it has become clear that a resilience process is common, can be expressed throughout different pathways and results from the interplay between intrinsic and extrinsic resources [8-10].

During the last decades, the focus has shifted from children dealing with chronic conditions to adults confronted with a single, potentially traumatic event (PTE). Resilience has been studied, for instance, in victims of terrorist attacks [11], in patients confronted with fatal illness [12] and during the bereavement period [13]. A PTE refers to a rather exceptional, aversive event which can evoke resilience, whether it is experienced traumatically or not [14]. Resilience was no longer considered a trait but rather the process of coping with adversity leading to fortification of the resiliency characteristics [2].

# References

1. Seligman ME, Csikszentmihalyi M. Positive psychology. An introduction. Am Psychol. 2000 Jan;55(1):5-14.

2. Richardson GE. The metatheory of resilience and resiliency. J Clin Psychol. 2002 Mar;58(3):307-321.

3. Scoloveno R. A concept analysis of the phenomenon of resilience. J nurs care. 2016;5(4).

4. Masten AS. Ordinary magic. Resilience processes in development. Am Psychol. 2001 Mar;56(3):227-38.

5. Garmezy N. Resilience in children's adaptation to negative life events and stressed environments. Pediatr Ann. 1991 Sep;20(9):459-460, 463-466.

6. Rutter M. Psychosocial resilience and protective mechanisms. Am J Orthopsychiatry. 1987 Jul;57(3):316-331.

7. Werner E, Smith R. Overcoming the odds: high risk children from birth to adulthood. New York: Cornell University Press; 1992.

8. Bonanno GA. Loss, trauma, and human resilience: have we underestimated the human capacity to thrive after extremely aversive events? The American Psychologist. 2004 Jan;59(1):20-28.

9. Bonanno GA. Resilience in the face of potential trauma. Curr Dir Psychol Sci. 2005;14(3):135-138.

10. Lepore SJ, Revenson TA. Resilience and posttraumatic growth: recovery, resistance, and reconfiguration. In: Calhoun LG, Tedeschi RG, editors. Handbook of Posttraumatic Growth Research and Practice. Mahwah, New Jersey (NJ) 2006. p. 24-46.

11. Bonanno GA, Galea S, Bucciarelli A, et al. Psychological resilience after disaster: New York City in the aftermath of the September 11th terrorist attack. Psychol Sci. 2006 Mar;17(3):181-186.

12. Folkman S, Greer S. Promoting psychological well-being in the face of serious illness: when theory, research and practice inform each other. Psycho-oncology. 2000 Jan-Feb;9(1):11-9.

13. Bonanno GA, Moskowitz JT, Papa A, et al. Resilience to loss in bereaved spouses, bereaved parents, and bereaved gay men. J Personal Social Psychol. 2005 May;88(5):827-843.

14. Bonanno GA, Mancini AD. The human capacity to thrive in the face of potential trauma. Pediatrics. 2008 Feb;121(2):369-375.
