# Supplementary material for: Resilience in family caregivers of patients diagnosed with advanced cancer – unravelling the process of bouncing back from difficult experiences, a hermeneutic review
Source: Eur J Gen Pract. 2020 Jul 7;26(1):79–85. doi: 10.1080/13814788.2020.1784876 (PMC7470057; doi:10.1080/13814788.2020.1784876)
Supplement: Supplemental Material - Conceptualization of resilience [file IGEN_A_1784876_SM1281.docx]

Conceptualization of resilience

Resilience has been conceptualised in different ways.

#### Resilience as a Trait: Ego-Resiliency

Earlier research has mainly addressed resilience as a personality trait, mostly referred to as resiliency [1] or ego-resiliency [2, 3].

In special editions of *the American Psychologist* and *the Journal of Social and Clinical Psychology*, resilient qualities such as optimism, faith, wisdom, creativity, forgiveness, gratitude or self-control have been described extensively [1]. It cannot be denied that the identification of resilient qualities has contributed significantly to the insights into how people adapt to new circumstances [1]. Nevertheless, the term ‘a resilient person’ only refers to a person’s individual resilience resources and does not guarantee a resilient process when confronted with adversity [4].

#### Resilience as a Biopsychospiritual Homeostasis

Flach (1997) and Richardson (2002) advocate that resilient qualities could be attained through repetitive disruptions [1,5]. In his resiliency theory, Richardson studies people who can depend upon sufficient resilient qualities detecting opportunities to further develop from adversity, and hence, reintegrate resiliently. He describes how the interaction between the protective, resilient qualities and the threats coming with adversity can lead to biopsychospiritual homeostasis on the one hand or to dysfunction in people who lack resilient qualities on the other [1].

#### Resilience as a Dynamic Process

Lately, most experts agree that resilience is a common phenomenon [6-13]. It is a process resulting from the interplay between the human capacity to adapt to adversity and the behaviour within the context. Resilience is a dynamic process that cannot be considered separately from the potentially traumatic event (PTE) or stressor [14]. Table 2 presents descriptions of the theoretical frameworks resulting from concept analyses on resilience following a PTE.

# References

1. Richardson GE. The metatheory of resilience and resiliency. J Clin Psychol. 2002 Mar;58(3):307-321.

2. Luthar SS, Cicchetti D, Becker B. The construct of resilience: a critical evaluation and guidelines for future work. Child Dev. 2000 May-Jun;71(3):543-562.

3. Letzring TD, Block J, Funder DC. Ego-control and ego-resiliency: generalization of self-report scales based on personality descriptions from acquaintances, clinicians, and the self. J Res Personal. 2005;39:395-422.

4. Bonanno GA. Uses and abuses of the resilience construct: loss, trauma, and health-related adversities. Soc Sci Med (1982). 2012 Mar;74(5):753-756.

5. Flach FF. Resilience: how to bounce back when the going gets tough? New York (NY): Hatherleigh Press; 1997.

6. Eicher M, Matzka M, Dubey C, et al. Resilience in adult cancer care: an integrative literature review. Oncol Nurs Forum. 2015 Jan;42(1):3-16.

7. Bonanno GA. Resilience in the face of potential trauma. Curr Dir Psychol Sci. 2005;14(3):135-138.

8. Bonanno GA. Loss, trauma, and human resilience: have we underestimated the human capacity to thrive after extremely aversive events? The American psychologist. 2004;59(1):20-8.

9. Bonanno GA, Kennedy P, Galatzer-Levy IR, et al. Trajectories of resilience, depression, and anxiety following spinal cord injury. Rehabil Psychol. 2012 Aug;57(3):236-247.

10. Bonanno GA, Malgaroli M. Trajectories of grief: Comparing symptoms from the DSM-5 and ICD-11 diagnoses. Depress Anxiety. 2019 Apr 22.

11. Bonanno GA, Westphal M, Mancini AD. Resilience to loss and potential trauma. Ann Rev Clin Psychol. 2011;7:511-535.

12. Southwick SM, Bonanno GA, Masten AS, et al. Resilience definitions, theory, and challenges: interdisciplinary perspectives. Eur J Psychotraumatol. 2014;5.

13. Mancini AD, Sinan B, Bonanno GA. Predictors of prolonged grief, resilience, and recovery among bereaved spouses. J Clin Psychol. 2015 Dec;71(12):1245-1258.

14. Bonanno GA, Romero SA, Klein SI. The temporal elements of psychological resilience: an integrative framework for the study of individuals, families, and communities. Psychol inq. 2015;26(2):139-169.
