# Supplementary material for: Resilience in family caregivers of patients diagnosed with advanced cancer – unravelling the process of bouncing back from difficult experiences, a hermeneutic review
Source: Eur J Gen Pract. 2020 Jul 7;26(1):79–85. doi: 10.1080/13814788.2020.1784876 (PMC7470057; doi:10.1080/13814788.2020.1784876)
Supplement: Supplemental Material - Author contributions [file IGEN_A_1784876_SM1280.docx]

Background and expertise of the authors and their contribution to the review.

| Author initials | Profession | Expertise | Contribution to the review | | | | | | |
| --- | --- | --- | --- | --- | --- | --- | --- | --- | --- |
|  |  |  | Study assignment | Design and methodology | Data extraction and analysis | Writing- original draft | Writing- review and editing | Validation of the final version | Supervision |
| SO | PhD student; GP | Palliative care  Qualitative research |  | X | X | X |  | X |  |
| JDL | Professor in Primary Care; GP | Palliative Care; Communication in health care | X |  | X |  | X | X |  |
| EL | Professor in clinical psychology | Research on behavioural change and health communication |  |  | X |  | X | X |  |
| PP | Professor in Primary Care; GP | Palliative care  Research on communication in health care and complexity science | X | X | X |  | X | X | X |
