# Supplementary material for: Resilience in family caregivers of patients diagnosed with advanced cancer – unravelling the process of bouncing back from difficult experiences, a hermeneutic review
Source: Eur J Gen Pract. 2020 Jul 7;26(1):79–85. doi: 10.1080/13814788.2020.1784876 (PMC7470057; doi:10.1080/13814788.2020.1784876)
Supplement: Supplemental Material - Theoretical frameworks of resilience [file IGEN_A_1784876_SM1279.docx]

Theoretical frameworks of resilience resulting from concept analyses designed to study resilience following PTE.

| Author | Year | Concept/framework name | Description |
| --- | --- | --- | --- |
| Richardson GE [44]. | 2002 | The Resiliency Model | People have the opportunity to choose consciously or unconsciously the outcome of disruptions. Resiliency starts with biopsychospiritual homeostasis reached through adaptation to earlier adversity or stressful live events. New disruptions can lead to: 1) resilient reintegration, meaning that the resilient qualities are strengthened and growth is experienced; 2) turning down opportunities for growth and healing by reintegration back to homeostasis; 3) recovering with loss of hope and motivation; or 4) dysfunctional reintegration often accompanied by dysfunctional behaviour. |
| Agaibi CE. & Wilson JP. [50] | 2005 | A generic model of resilience to psychological trauma | Traumatic life experiences evoke behaviour determined by the complex interaction of key variables on different levels (e.g., coping styles, affect modulation, personality characteristics, locus of control, ego-defensive processes and protective factors). The variables can work together to produce different degrees of resilience or adaptive behaviour on a continuum from high resilience and optimal adaptive coping to low resilience with risk for psychopathology. |
| Gillespie BM. et al. [51] | 2007 | A theoretically derived model of resilience | Resilience is conceptualized by its antecedents (adversity, the situation being interpreted as traumatic, the cognitive ability to interpret adversity and a realistic worldview), its defining attributes (self-efficacy, hope and coping) and its consequences (integration in context, development of control, psychological adjustment and personal growth). |
| Davydov DM. et al. [16] | 2010 | A biopsychosocial (multi-level) construct for mental resilience | Health is protected by two similar concepts, namely the somatic immune system and the mental resilience system. Resilience mechanisms recognize threat and neutralize adversity. Part of the mechanisms are innate, others are developed naturally through by adaptation or by external influences. The mechanisms may interact with each other or even constitute a causal chain, leading to protection of mental health, recovery or reduction of negative effects of stressors. |
| Windle G. [33] | 2011 | Requirements for resilience | Resilience requires three essential factors: firstly, there should be significant adversity; secondly, resources or assets are needed to reduce the effects of adversity; and finally, a negative outcome should be avoided by positive adaptation. |
| Garcia-Dia MJ. [36] | 2013 | Resilience concept mapping | Resilience is precipitated by personality traits, experiences and internal or external factors. Those factors can either protect and lead to resilience or place the individual at risk and lead to maladaptation. Resilience is a process that surfaces from within or develops through adversity, resulting in effective coping (e.g., redefining goals, recovering physically and psychologically and reaching personal growth or spirituality) and re-integrating into society by rebounding, determination, self-efficacy and social support. |
| Bonanno GA. et al. [49] | 2015 | The temporal elements of psychological resilience | Resilience is a broad, umbrella phenomenon that encompasses four temporally related elements: 1) baseline, pre-adversity adjustment; 2) aversive circumstances; 3) post-adversity resilient outcomes; and 4) predictors of resilient outcomes evolving in the course of the resilient trajectory. |
| Liu JJW. et al. [52] | 2017 | Multi-system model of resilience (MSMR) | A multi-system model consisting of three layers: 1) core resilience existing of trait-like characteristics; 2) internal resilience comprising personality developed or acquired over time through experiences and social interactions; and 3) external or community resilience, situating each individual in a larger socio-economic context. |

Legend: The description of the theoretical frameworks are summarizations of the research findings in the concept analyses. All frameworks are presented graphically in the original papers. The numbers [ ] refer to the references in the main text.
