# Supplementary material for: Resilience in family caregivers of patients diagnosed with advanced cancer – unravelling the process of bouncing back from difficult experiences, a hermeneutic review
Source: Eur J Gen Pract. 2020 Jul 7;26(1):79–85. doi: 10.1080/13814788.2020.1784876 (PMC7470057; doi:10.1080/13814788.2020.1784876)
Supplement: Supplemental Material - Definitions of resilience [file IGEN_A_1784876_SM1278.docx]

Definitions of resilience following a PTE, in cancer care, and caregivers. The definitions are cited from systematic reviews, concept analyses and other review papers.

| Author | Title | Year | Definition of resilience |
| --- | --- | --- | --- |
| Resilience following a PTE | | | |
| Bonanno GA. [2] | Loss, trauma, and human resilience. Have we underestimated the human capacity to thrive after extremely aversive events? | 2004 | Resilience reflects the ability to maintain a stable equilibrium. It pertains to the ability of adults in otherwise normal circumstances who are exposed to an isolated and potentially highly disruptive event, to maintain relatively stable, healthy levels of psychological and physical functioning. Resilience is more than the simple absence of psychopathology. |
| Letzring TD. et al. [29] | Ego-control and ego-resiliency: generalisation of self-report scales based on personality descriptions from acquaintances, clinicians, and the self | 2005 | Ego-resiliency is conceptualised as a central personality construct for understanding motivation, emotion and behaviour. It refers to a meta-dimension of the dynamic capacity to contextually modify one’s level of control in response to situational demands and affordances. |
| Bonanno GA. [28] | Resilience in the face of potential trauma | 2005 | Resilience is characterised by relatively mild and short-lived disruptions and a stable trajectory of healthy functioning across time. |
| Yehuda R. et al. [12] | Developing an agenda for translational studies of resilience and vulnerability following trauma exposure | 2006 | Resilience is the process of adapting well in the face of adversity, trauma, tragedy, threats of harm or even significant sources of stress. Psychological resilience can be viewed as a relatively stable constitutional resource characterized by the ability to bounce back from a negative experience or even significant adversity by flexible adaptation to the ever-changing demands of life. |
| Bonanno GA. & Mancini AD. [30] | The human capacity to thrive in the face of potential trauma | 2008 | Resilience to potential trauma is the ability of adults in otherwise normal circumstances who are exposed to an isolated and potentially highly disruptive event such as the death of a close relation or a violent or life-threatening situation to maintain relatively stable, healthy levels of psychological and physical functioning, as well as the capacity for generative experiences and positive emotions. |
| Grafton E. et al. [31] | Resilience: the power within | 2010 | The ability to cope with or recover from the impact of stress and turn it into a positive learning experience is described as resilience. |
| Bonanno GA. et al. [32] | Resilience to loss and potential trauma | 2011 | Resilience is a stable trajectory of healthy functioning following a potentially traumatic event. Resilience is characterized by relatively minor and transient disruptions in functioning, with few if any marked effects on everyday functioning and routines. |
| Windle G. [33] | What is resilience? A review and concept analysis | 2011 | Resilience is the process of effectively negotiating, adapting to, or managing significant sources of stress or trauma. Assets and resources within the individual, their life and environment facilitate this capacity for adaptation and ‘bouncing back’ in the face of adversity. Across the life course, the experience of resilience will vary. |
| Bonanno GA. [34] | Uses and abuses of the resilience construct: Loss, trauma and health-related adversities | 2012 | Resilience is a stable trajectory of healthy functioning in response to a clearly defined event. |
| Rutter M. [35] | Resilience as a dynamic concept | 2012 | Resilience can be defined as reduced vulnerability to environmental risk experiences, the overcoming of stress or adversity or a relatively good outcome despite risk experiences. It is an interactive concept in which the presence of resilience has to be inferred from individual variations in outcome among individuals who have experienced significant major stress or adversity. |
| Garcia-Dia MJ. [36] | Concept analysis: resilience | 2013 | Resilience is one’s ability to bounce back or recover from adversity. It is a dynamic process that can be influenced by the environment, external factors and/or the individual and the outcome. |
| Hu T. et al. [37] | A meta-analysis of the trait resilience and mental health | 2015 | Trait resilience is a personal trait that helps individuals cope with adversity and achieve proper adjustment and development. It is a personality trait that inoculates individuals against the impact of adversity or traumatic events. |
| Seery MD. & Quinton WJ. [24] | Understanding resilience: from negative life events to everyday stressors | 2016 | Resilience reflects managing well with stressors in general. |
| Kalisch R. et al. [38] | The resilience framework as a strategy to combat stress-related disorders | 2017 | Resilience is the maintenance or quick recovery of mental health during and after exposure to significant stressors and results from a dynamic process of adaptation to the given stressful life circumstances. Resilience is not merely inertia or insensitivity to stressors or merely a passive response to adversity, but the result of active, dynamic adaptation. |
| Galatzer-Levy IR. et al. [39] | Trajectories of resilience and dysfunction following potential trauma: A review and statistical evaluation | 2018 | Minimal impact resilience is a process characterized by stable psychological and physical health from before to after the PTE. |
| Resilience in caregivers | | | |
| Rosa F et al. [40] | Resilience as a concept for understanding family caregiving of adults with chronic obstructive pulmonary disease (COPD): an integrative review | 2016 | Resilience in COPD caregivers can be described as an enduring ability or capacity that is exhibited as a strength of the caregiver when responding to acute exacerbations, chronic stresses and problem solving for symptoms management and which can be further developed. |
| Autio T. & Rissanen S. [41] | Positive emotions in caring for a spouse: a literature review | 2017 | Resilience is a ‘bounce back’ or return to prior or normal functioning after facing difficulties in life. It contains thoughts, behaviour and actions that anyone can learn, and it is not a permanent trait of personality that you either have or do not have. |
| Resilience in cancer care | | | |
| Eicher M. et al. [42] | Resilience in adult cancer care: an integrative literature review | 2015 | Resilience in adult patients with cancer and in cancer survivors is a dynamic process of facing adversity related to the cancer experience. |

Legend: The definitions are reproduced verbatim from the reviews. The numbers refer to the references presented in the main text.
